# Inflammaging: Experimental Insights and Translational Advances

**DOI:** 10.1002/eji.70239

**Published:** 2026-07-26

**Authors:** Andrea Cossarizza

**Affiliations:** ^1^ Chair of General Pathology and Immunology University of Modena and Reggio Emilia School of Medicine Modena Italy

**Keywords:** clinical trial, immune system, inflammasome, inflammation, innate immune system, medicine, reproductive senescence, senescence

## Abstract

Inflammaging, defined as the persistent, low‐grade sterile inflammation accompanying aging, represents a central driver of age‐related pathology, including cardiovascular dysfunction, neurodegeneration, metabolic disorders, and frailty. This review discusses the most recent advances in understanding its mechanistic basis, encompassing cellular senescence, the senescence‐associated secretory phenotype (SASP), mitochondrial dysfunction, immune cell senescence, innate immune hyperactivation, defective inflammatory resolution, and nutrient‐sensing dysregulation. Single‐cell and spatial transcriptomics reveal tissue‐specific and context‐dependent patterns, highlighting the systemic complexity of inflammaging. Preclinical interventions demonstrate that inflammaging is modifiable through senolytics, which selectively eliminate senescent cells, and senomorphics, which suppress SASP without inducing cell death. Metabolic modulators such as metformin and rapamycin attenuate inflammatory signaling, while immune‐directed therapies and microbiome‐targeted interventions provide synergistic benefits through combinatorial approaches. Early‐phase clinical trials in frail older adults show feasibility, safety, and preliminary efficacy, including reductions in circulating inflammatory markers, improved physical function, and enhanced immune responsiveness. Inflammaging trajectories are shaped by lifestyle, environmental exposures, and evolutionary factors, underscoring the need for personalized interventions. Remaining challenges include biomarker development, long‐term safety evaluation, and heterogeneity across aging populations. Addressing these through interdisciplinary research supports a precision geroscience paradigm, where multimodal targeting of inflammaging can extend healthspan and reduce chronic disease burden.

## Graphical abstract

Inflammaging is a chronic, low‐grade, sterile inflammation associated with aging that contributes to age‐related diseases and functional decline. Recent research suggests that targeting inflammaging through senolytics, metabolic modulators, microbiome‐based interventions, and lifestyle modifications may help improve healthspan, reduce chronic disease burden, and promote healthy aging.

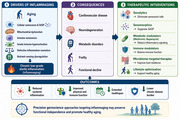

## 1 Introduction

The concept of inflammaging describes the paradoxical increase of inflammatory phenomena and systemic mediators in elderly individuals [1, 2]. This low‐grade, chronic inflammation is linked to frailty, multimorbidity, and increased mortality, highlighting inflammation as a key driver of age‐related decline [3, 4]. Evolutionarily, inflammaging reflects antagonistic pleiotropy: early‐life valid immune activation provides protection and reproductive fitness, but persistent inflammation in later life promotes tissue damage and chronic disease [5, 6].

Epidemiological studies show that elevated IL‐6, TNF, and C‐reactive protein (CRP) predict frailty, sarcopenia, cognitive decline, and cardiovascular events, independent of traditional risk factors [7, 8]. Mechanistically, a substantial contribution comes from the senescence‐associated secretory phenotype (SASP), a key biological feature of senescent cells (described below) [9].

Inflammaging is supposed to be a main causal driver of multiple age‐related diseases, including cardiovascular disease, neurodegeneration, metabolic syndrome, osteoporosis, and cancer [10, 11]. Chronic activation of innate immune pathways, such as those of NF‐κB and NLRP3, along with adaptive immune dysregulation, further exacerbates the inflammatory burden [12].

Over the past few years, research has increasingly focused on tissue‐specific drivers, including the gut microbiome, adipose tissue, and vascular endothelium. Single‐cell transcriptomics and multi‐omics approaches reveal heterogeneity in inflammaging across organs and individuals [13]. Preclinical studies demonstrate that interventions targeting senescent cells, such as senolytics or metabolic modulators, can reduce systemic inflammation and improve tissue function, with early human studies showing promise for mitigating frailty and chronic disease markers [14, 15, 16].

It is to note that the COVID‐19 pandemic has revealed profound interactions between viral infection and inflammaging. SARS‐CoV‐2 infection triggers an intense innate immune response characterized by hyperactivation of NF‐κB, NLRP3 inflammasome, and cGAS–STING pathways, precipitating a massive immune activation and a cytokine milieu that parallels and amplifies chronic low‐grade inflammation [17]. Importantly, COVID‐19 induces widespread cellular senescence across multiple tissues, resulting in an enhanced SASP that perpetuates systemic inflammation and tissue dysfunction. Concurrently, the virus exacerbates immunosenescence by depleting naïve lymphocytes, expanding exhausted T cell populations, and altering the B cell compartment [18, 19], thereby undermining immune surveillance and promoting persistent inflammation, especially in aged patients [20]. Endothelial dysfunction, mitochondrial impairment, and disruption of autophagic and antioxidant pathways in innate immune cells further contribute to a pro‐inflammatory metabolic milieu [21]. Alterations in the gut microbiome and sustained innate immune activation sustain this chronic inflammatory state, which manifests clinically in long COVID as a persistent inflammaging phenotype. Collectively, these convergent mechanisms accelerate biological aging and exacerbate age‐associated pathologies.

Thus, inflammaging is a multifactorial process that integrates not only core biological hallmarks of aging, such as cellular senescence, immune dysregulation, metabolic alterations, and impaired resolution of inflammation, but also the organism's response to the exposome [22]. The exposome, defined as the cumulative measure of all environmental and lifestyle exposures across the lifespan, profoundly influences biological aging, healthspan, and vulnerability to age‐related diseases. It encompasses both external factors, including pollutants, diet, infections, and psychosocial stress, and internal processes such as metabolic activity, microbiome dynamics, oxidative stress, and chronic inflammation. Understanding the mechanisms driving inflammaging and the factors contributing to its interindividual variability is essential for designing interventions aimed at extending healthspan and preventing age‐related diseases. Such insights may herald a paradigm shift in aging therapeutics.

## 2 Molecular and Cellular Foundations of Inflammaging

**FIGURE 1:**
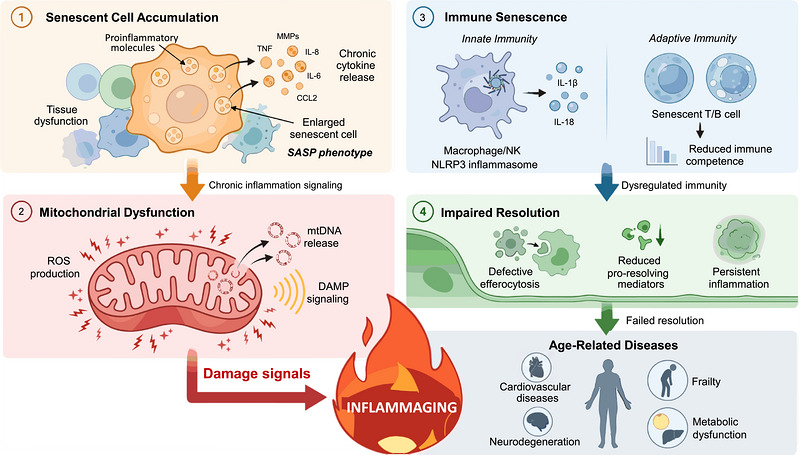
Molecular and cellular foundations of inflammaging. Cellular senescence, mitochondrial dysfunction, innate and adaptive immune senescence, and impaired resolution of inflammation are major drivers of inflammaging. Through the production of pro‐inflammatory mediators, activation of innate immune pathways, accumulation of dysfunctional immune cells, and failure to restore tissue homeostasis, these interconnected mechanisms promote chronic low‐grade inflammation and contribute to age‐related diseases and functional decline.

As schematically illustrated in Figure 1, cellular senescence, mitochondrial dysfunction, innate and adaptive immune senescence, and impaired resolution of inflammation are major drivers of inflammaging. Through the production of pro‐inflammatory mediators, activation of innate immune pathways, accumulation of dysfunctional immune cells, and failure to restore tissue homeostasis, these interconnected mechanisms promote chronic low‐grade inflammation and contribute to age‐related diseases and functional decline.

### 2.1 Cellular Senescence

Cellular senescence induces the so‐called SASP, characterized by the production of a heterogeneous mixture of cytokines, chemokines, growth factors, and proteases, including IL‐6, IL‐1β, TNF, and matrix metalloproteinases [23]. SASP can support tissue repair and tumor suppression, but also promotes chronic inflammation and tissue dysfunction [24]; its composition is highly tissue‐specific and depends on cell type, microenvironment, and stressor exposure. Recent single‐cell analyses have revealed heterogeneity among senescent cells within the same tissue, showing subpopulations with pro‐inflammatory or immunomodulatory profiles, highlighting the need for targeted interventions [25].

### 2.2 Mitochondrial Dysfunctions

Mitochondrial dysfunctions contribute to inflammaging through excess reactive oxygen species (ROS) production and release of mitochondrial DNA (mtDNA), which acts as a damage‐associated molecular pattern (DAMP) activating innate immune receptors [26]. Impaired mitophagy exacerbates oxidative stress and inflammation, creating a self‐reinforcing cycle [27] that is well revealed by the age‐dependent increase in plasma levels of mtDNA [28]. Strategies that enhance mitochondrial quality control, including exercise, NAD+ precursors, and pharmacologic activators of mitophagy, have shown efficacy in preclinical models in reducing chronic inflammation [29].

### 2.3 Innate and Adaptive Senescence

It is well known that innate immune cells, including macrophages and dendritic cells, exhibit hyperactivation with age, with NLRP3 inflammasome and NF‐κB signaling elevating IL‐1β and IL‐18 production. Age‐related defects in pathogen clearance further exacerbate the pro‐inflammatory milieu. Adaptive immunity is also modified with age: thymic involution diminishes naïve T cell output, skewing the T cell repertoire [30, 31], while senescent T cells secrete SASP‐like factors that reinforce systemic inflammation. Also, aging B cells demonstrate reduced antibody affinity and impaired class switching, contributing to weaker humoral responses [32].

### 2.4 Impaired Resolution of Inflammation

The resolution of inflammation is due to the biosynthesis and bioavailability of specialized pro‐resolving mediators (SPM), such as resolvins, protectins, maresins, and lipoxins [33]. These lipid‐derived mediators play a critical role in orchestrating the active termination phase of inflammation by promoting the cessation of neutrophil infiltration, enhancing macrophage‐mediated apoptotic cell clearance (defined as efferocytosis) of apoptotic cells, and facilitating tissue repair and homeostasis restoration. It has been hypothesized that they also might have a role in the control of inflammation in aged individuals [34]. Indeed, it has been shown that old mice have reduced levels of SPM, which lead to a failure in these pro‐resolving pathways, resulting in a sustained and dysregulated inflammatory milieu [35].

Concurrently, aging is associated with a decline in the efficiency of efferocytosis and an accumulation of senescent cells, which resist apoptosis and assume a SASP. The inefficient removal of these cellular debris and senescent cells further exacerbates inflammation by continuously stimulating innate immune receptors and perpetuating cytokine release. Thus, impaired SPM production coupled with defective clearance mechanisms forms a feed‐forward loop that prolongs and intensifies chronic inflammation in aging tissues [36, 37].

In summary, inflammaging arises from the convergence of senescent cell accumulation, mitochondrial dysfunction, innate and adaptive immune remodeling, and impaired resolution. This complex network underlies multiple age‐related diseases, including cardiovascular disease, neurodegeneration, and frailty, and emphasizes the need for combinatorial interventions targeting multiple molecular pathways.

## 3 Experimental Models and Preclinical Breakthroughs

### 3.1 In Vitro and In Vivo Models

Experimental and translational models have been instrumental in deconvoluting the mechanistic architecture of inflammaging. Studies in murine models, including NLRP3‑ or IL‑1β‑knockout mice and strains carrying inducible senescent‑cell reporters (designed to detect, track, and manipulate senescent cells in vivo), have provided compelling evidence for causal links between senescence, SASP factors, and systemic inflammation [38, 39]. Clearance of p16^Ink4a^ senescent cells (lacking the tumor suppressor protein that inhibits CDK4/6, halting the cell cycle in the G1 phase) reduces pro‐inflammatory cytokines, improves tissue regeneration, and delays functional decline [40]. Tissue‐specific models show organ‐dependent contributions of senescent cells, with pronounced effects in liver, adipose tissue, and vasculature [41, 42].

Metabolic pathways integrate with inflammaging through nutrient‐sensing and stress‐response nodes. Modulation of mTOR, AMPK, and S6K1 pathways influences inflammatory responses [43]. Hepatic deletion of S6K1 reduces local and systemic inflammation without eliminating senescent cells, suggesting metabolic interventions can partially dissociate inflammation from senescence [44]. Caloric restriction (CR) and CR mimetics (resveratrol, spermidine, NAD+ boosters) attenuate inflammaging markers, decrease hypertension, and enhance lifespan in murine models [45, 46, 47].

Immune‐directed interventions act upstream of the inflammaging cascade. Clearance or functional reprogramming of senescent T, B, and myeloid populations restores immune surveillance and reduces systemic cytokine load. IL‐1β blockade, PD‐1/PD‐L1 modulation, and thymic rejuvenation strategies expand naïve T‐cell pools and mitigate chronic innate immune activation [48, 49]. When combined with senolytics, such interventions markedly enhance senescent cell clearance and potentiate anti‐inflammatory responses, although their translatability to humans remains to be fully evaluated.

The gut microbiome operates as a critical rheostat of the inflammaging network. Microbiome‐directed interventions based upon antibiotic conditioning, probiotic reconstitution, and fecal microbiota transfer can reshape microbial‐derived metabolites, TLR ligands, and inflammasome‐activating signals, thereby modulating systemic SASP transcriptional programs and inflammasome tone [50]. Human organoids and 3D human‐immune co‐cultures recapitulate senescence‐immune‐microbiome interactions with clinically relevant fidelity, enabling mechanistic dissection and high‐throughput intervention testing [51].

### 3.2 Effects of Senolytics or Senomorphics in Different Settings

Senolytics, including dasatinib plus quercetin, fisetin, navitoclax derivatives, and emerging Bcl‐xL–sparing analogs, are able to induce apoptosis in senescent cells by exploiting their dependency on pro‐survival networks such as Bcl‐2/Bcl‐xL, PI3K/Akt, and FOXO‐mediated anti‐apoptotic programs [52, 53]. Their systemic effects include suppression of SASP cytokines, restoration of vascular compliance, enhancement of mitochondrial function, and improvements in neurocognitive and cardiometabolic performance. Tissue‐specific senolysis, delivered through nanoparticle formulations or ligand‐directed approaches, increases selectivity for adipose, vascular, or skeletal muscle compartments, improving safety profiles while intensifying local SASP reduction [54, 55, 56].

Senomorphics modulate SASP transcriptional programs without eliminating cells [57]. JAK/STAT inhibitors dampen IL‐6/IL‐8/MCP‐1 secretion; mTOR inhibitors such as rapamycin suppress SASP via reduced NF‐κB activation and enhanced autophagy; and metformin decreases mitochondrial ROS and cGAS–STING activation, attenuating type I interferon responses. Combinatorial senolytic–senomorphic strategies display synergistic repression of the SASP while limiting off‐target cytotoxicity [58], reflecting the layered regulatory architecture of senescence‐associated inflammatory programs.

Tissue‐specific interventions are critical: adipose‐targeted senolytics improve metabolic health [59]. Vascular‐targeted “senotherapeutics” alleviate endothelial dysfunction and arterial stiffness by clearing senescent endothelial and smooth muscle cells, restoring nitric oxide signaling and vascular compliance [60]. Concurrently, skeletal muscle‐targeted clearance of senescent progenitors enhances regenerative capacity and mitigates sarcopenia, offering a dual‐compartment strategy for age‐related cardiovascular and musculoskeletal decline [61]. Mechanistic studies using single‐cell transcriptomics and proteomics reveal selective modulation of fibroblasts, endothelial cells, and immune subsets, while biomarker identification supports monitoring and patient selection.

### 3.3 The “OMICS” and the Biological Clocks

At the systems level, multi‐omics integration (i.e., single‐cell transcriptomics, proteomics, metabolomics, lipidomics, methylomics, among others) reveals conserved network modules linking metabolic dysfunction, innate immune hyperactivation, mitochondrial stress, epigenetic drift, and microbiome‐derived inflammatory cues. These analyses highlight the inherently multinodal, feed‐forward architecture of inflammaging, suggesting that durable mitigation will require modular combination therapies spanning senolytic, senomorphic, metabolic, and immune‐targeted axes. Collectively, preclinical advances in recent years delineate a coherent translational trajectory toward interventions that extend healthspan by dismantling the molecular and cellular circuitry sustaining chronic age‐associated inflammation. These preclinical breakthroughs provide a foundation for human translation, suggesting that modular, multitargeted strategies can mitigate inflammaging, improve healthspan, and prevent age‐related diseases.

Genome‐wide association studies (GWAS) have identified variants in genes encoding pro‐inflammatory cytokines, most notably IL‐6, IL‐1β, TNF, and NF‐κB pathway components that associate with longevity and disease susceptibility. Franceschi et al. first framed inflammaging as a progressive reshaping of inflammatory mediator expression driven in part by chronic antigenic load and genetic predisposition [62]. They proposed that methylation, glycomics, metabolomics, and lipidomics are useful biomarkers capable of assessing biological versus chronological age in metabolic diseases and connected gut microbiota to both metaflammation and inflammaging.

**FIGURE 2:**
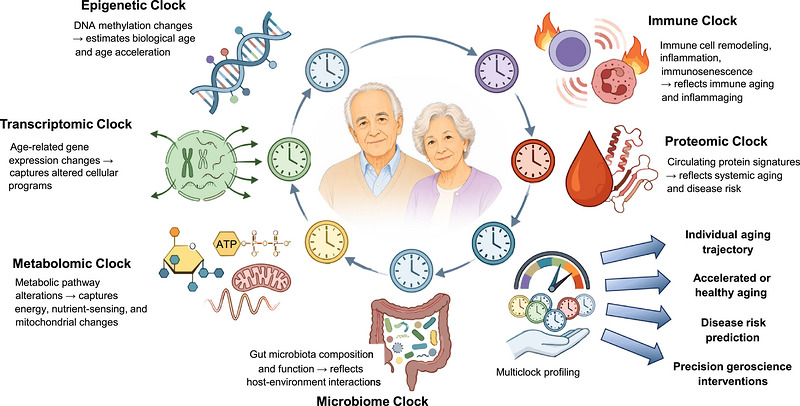
Biological clocks provide complementary information on immune aging. Some of them reflect cellular and molecular damage, others capture immune dysfunction, epigenetic changes, metabolic alterations, or environmental influences. Integrating multiple biological clocks may therefore offer a more accurate picture of individual aging trajectories, helping to identify accelerated aging, predict disease risk, and guide precision interventions aimed at promoting healthy aging.

DNA methylation is the most thoroughly studied epigenetic layer in aging. Epigenetic aging clocks such as PhenoAge, GrimAge, and DunedinPoAm are widely studied to capture the real biological age of a person [63]. They are able to estimate biological age from DNA methylation patterns and are among the most widely used biomarkers of aging, along with immune clocks, that measure age‐related remodeling of the immune system, including changes in lymphocyte subsets, inflammation, and immunosenescence. Transcriptomic, proteomic, metabolomic, and microbiome‐based clocks capture additional molecular and functional changes that occur with age (Figure 2). Integrative transcriptome and epigenome analyses (RNA‐seq + ATAC‐seq) have revealed that enhanced inflammatory response is the dominant common signature of aging across tissues, with AP‐1 transcription factors as key regulators. Chromatin accessibility studies have further shown that NF‐κB and AP‐1 binding sites become progressively more open with age, providing a mechanistic link between epigenomic remodeling and sustained low‐grade inflammation [64, 65].

Bulk RNA‐seq has been instrumental in mapping tissue‐specific inflammaging signatures. A comprehensive transcriptomic analysis of 40 human tissues from the GTEx project identified over 17,000 differentially expressed genes between individuals under 40 and over 65, with upregulated genes enriched for inflammation, immune responses, and apoptosis, and downregulated genes linked to mitochondrial function [66]. Single‐cell transcriptomics has added cell‐type resolution and precious information. Clonal GZMK^+^ CD8^+^ T cells are a conserved hallmark of inflammaging [67], and peripheral blood mononuclear cells were RNA‐sequenced at the single‐cell level to develop aging clocks, revealing that the ribosome‐to‐inflammation balance is a single‐cell aging hallmark even in supercentenarians [68]. More recently, sc‐ImmuAging clocks, trained on scRNA‐seq data from 1081 healthy individuals aged 18–97, demonstrated cell‐type‐specific age acceleration in monocytes during COVID‐19 infection, with partial recovery during convalescence [69].

Large‐scale proteomic studies have fundamentally reshaped our understanding of human aging by demonstrating that chronic low‐grade inflammation is not merely a consequence of aging but one of its most pervasive molecular signatures. Across diverse cohorts, tissues, and analytical platforms, age‐associated changes in the proteome consistently converge on inflammatory, innate immune, complement, and senescence‐related pathways [70]. These findings support the concept of inflammaging as a systemic process arising from the cumulative effects of cellular senescence, immune dysregulation, tissue damage, and impaired resolution of inflammatory responses. Importantly, inflammatory proteins present in plasma represent some of the strongest predictors of biological age, frailty, multimorbidity, and mortality, highlighting their value as both mechanistic mediators and clinically relevant biomarkers of aging [71, 72]. Proteomic biomarkers capture inflammaging and immunosenescence features, including degradation of the extracellular matrix, fibrosis, and oxidative stress, that are not fully captured by DNA methylation clocks alone, making proteomics a complementary layer in multi‐omics organ clock frameworks [73].

Metabolomics has emerged as one of the most powerful approaches for studying inflammaging, as metabolites represent the downstream products of cellular activity and provide a functional readout of the interaction between metabolism, immunity, and aging. Unlike genomics or transcriptomics, metabolomics directly reflects physiological states and therefore captures the systemic metabolic consequences of chronic low‐grade inflammation that characterize aging [74]. Large‐scale metabolomic studies have consistently demonstrated that aging is associated with profound alterations in amino acid metabolism, lipid metabolism, mitochondrial function, redox balance, and immune‐metabolic pathways. Among the most reproducible findings are changes in tryptophan metabolism, accumulation of kynurenine pathway metabolites, altered branched‐chain amino acids, disturbances in NAD+ metabolism, and increased levels of metabolites associated with oxidative stress and chronic inflammation. Elevated circulating kynurenine levels correlate with increased concentrations of pro‐inflammatory cytokines such as TNF and IL‐6 and are associated with frailty, reduced muscle strength, impaired mobility, and adverse health outcomes in older adults, suggesting a mechanistic link between immune activation, altered tryptophan metabolism, and functional decline [75, 76].

Multi‐omics organ clocks integrating genomic, epigenomic, transcriptomic, proteomic, and metabolomic data are emerging as the most powerful tools to assess biological aging, capturing organ‐specific trajectories invisible to any single layer [73]. The use of artificial intelligence to integrate multiple omics with clinical parameters finally allows building organ‐specific aging clocks, able to predict the development of specific age‐related diseases [77]. In this regard, a web‐based database named exBAClock is available that integrates multiple functional modules considering >100 formulas from 95 publications [78]. Moreover, the term “ageotype” could be used to describe the different individual trajectories that might provide a novel tool for a personalized anti‐aging medicine [79].

## 4 Population and Evolutionary Perspectives

Inflammaging shows remarkable variability across populations, environments, and evolutionary histories, emphasizing its deeply context‐dependent nature, and indeed, age‐related inflammatory trajectories differ widely across human groups [80]. Indeed, researchers have recently examined whether a typical inflammaging axis—a cytokine‐based marker of aging identified in the Italian InCHIANTI study [81]—could be applied across different populations. An industrialized group of persons (Singapore Longitudinal Aging Study) was compared with two nonindustrialized groups (the Tsimane of Bolivia and the Orang Asli of Malaysia). The Singapore cohort largely mirrored the Italian findings, with minor differences in IL‑6 and IL‑1RA. The Tsimane and Orang Asli showed very different cytokine structures, with little or no link to aging or age‑related diseases. Moreover, despite the frequent exposure to infectious pathogens and the expected lack of antimicrobial therapies, they maintain low baseline levels of systemic inflammation [80]. Thus, inflammaging measured in this way appears to be a phenomenon tied mainly to industrialized lifestyles, not a universal biological process across all populations, indicating that lifestyle and environmental pressures, rather than aging itself, are the primary drivers of the “Western” inflammaging phenotype [82].

From an evolutionary standpoint, the concept of antagonistic pleiotropy provides a valuable framework for understanding why inflammaging emerges [83, 84]. The immune hyperactivation that protects young individuals from infections can become maladaptive later in life, promoting tissue damage and chronic inflammation as the body ages. Comparative research on long‐lived mammals, such as bats and naked mole rats, supports this view [85]. These species exhibit exceptionally low levels of systemic inflammation even in late life, reflecting unique immune regulatory mechanisms associated with extended longevity. The most compelling evidence supporting this phenomenon derives from observations in centenarians, who represent the most robust human model of exceptional longevity. These individuals exhibit a distinctly efficient anti‑inflammatory phenotype, as reflected by elevated plasma concentrations of multiple cytokines with established anti‑inflammatory activity, thereby indicating a finely tuned and highly effective immunoregulatory response [86]. Understanding how such mechanisms evolved may offer valuable clues for developing interventions that help humans modulate inflammaging more effectively.

A central modulator of population‐specific inflammatory patterns is the gut microbiome. Differences in microbial diversity and metabolite production are closely linked to systemic inflammatory profiles across populations [87]. Diet plays a major role in shaping this relationship, and it is well known that fiber‐rich diets that include fermented foods and plant‐based polyphenols tend to foster anti‐inflammatory microbial communities, whereas diets high in fat and refined sugar promote a pro‐inflammatory microbiome. Environmental influences, including exposure to chronic infections, air pollutants, psychosocial stressors, and social life, further shape immune reactivity, underscoring the complex interplay between biology, behavior, and ecology in determining the pace and intensity of inflammaging [88].

Inter‐individual heterogeneity in inflammaging reflects a complex interplay of allele‐specific modulation of innate immune signaling, chromatin architecture, and immunometabolic coupling. While a comprehensive analysis of the specific genes and haplotypes involved lies beyond the scope of this review, several illustrative examples underscore the mechanistic diversity underlying this phenomenon. Polymorphisms in key inflammasome components—such as *NLRP3*, *PYCARD* (ASC), *CASP1*, and *IL1B*—can alter supramolecular assembly kinetics by modulating PYD–CARD interaction affinities, ASC nucleation thresholds, and pro‐caspase‐1 autoproteolytic efficiency [89, 90]. These changes shift the basal stoichiometry of IL‐1β and IL‐18 maturation in aging myeloid cells, thereby influencing the amplitude and persistence of sterile inflammatory responses.

In parallel, promoter and enhancer variants in inflammatory genes, including *IL6*, *TNFA*, *IL10*, and *IFNB1*, reshape transcription factor occupancy, particularly of NF‐κB, AP‐1, and STAT3, and remodel histone landscapes marked by H3K27ac and H3K4me1 [91, 92]. These regulatory variants fine‐tune gene expression by altering chromatin accessibility and creating novel transcription factor binding sites, ultimately establishing stable, allele‐dependent inflammatory set points that interact with age‐associated epigenomic drift. Such genetic architectures converge with immunometabolic rewiring—characterized by mitochondrial DNA instability, cardiolipin remodeling, succinate‐driven HIF‐1α stabilization, and NAD^+^/SIRT axis decline—to shape the trajectory of inflammaging. Rather than representing a uniform or deterministic hallmark of aging, inflammaging emerges as a polygenic, network‐level phenotype governed by nonlinear interactions among germline variation, stochastic epigenetic remodeling, mitochondrial dysfunction, and chronic activation of pattern‐recognition pathways.

From a population and evolutionary perspective, inflammaging is best understood as a plastic and modifiable process. It arises from the dynamic integration of genetic predisposition, environmental exposures, microbiota composition, and lifestyle factors—and can therefore be reshaped through coordinated biomedical, behavioral, and public health interventions aimed at promoting healthy aging across diverse human populations.

## 5 Translational Advances: Clinical Studies

Recent advances in translational research have propelled anti‐inflammaging interventions from the realm of preclinical experimentation into early‐phase clinical trials, marking a pivotal transition toward modulating chronic low‐grade inflammation in humans and enabling more precise strategies to extend healthspan.

Among the most impactful approaches are senolytic therapies, engineered to selectively eliminate senescent cells by destabilizing the pro‐survival pathways that these cells rely on. Combinations such as dasatinib plus quercetin (D+Q), as well as the natural flavonoid fisetin, have generated encouraging early clinical signals both in nonhuman primates and in >50 years old patients with idiopathic pulmonary fibrosis [93, 94]. Pilot trials in frail older adults demonstrated reductions in systemic inflammatory cytokines such as IL‐6, TNF, and CRP, accompanied by improvements in muscle strength, gait velocity, endothelial function, and cardiorespiratory performance [95].

A recent pilot phase I study has assessed the effects of D+Q senolytic treatment on DNA methylation (DNAm), epigenetic age, and immune cell subsets in a group of donors. A second treatment group was added during the trial, which made use of D+Q and fisetin [96]. Notable differences were found in immune cell proportions between the DQ and DQ + fisetin treatment groups, providing a possible biological basis for the divergent patterns observed in the evolution of epigenetic clocks. However, re‐analyzing the impact of senolytic agents by using first‐, second‐, and third‐generation clocks revealed some incongruence, indicating that several tissue‐specific biomarkers are still needed to better understand the senolytic therapy effects.

Fisetin supplementation produced reductions in SASP‑associated cytokines and oxidative stress markers, consistent with conserved systemic senolytic activity across drug classes. Notably, intermittent dosing schedules appeared to optimize therapeutic indices by enhancing senescent‑cell apoptosis while minimizing cumulative toxicity, in agreement with preclinical pharmacokinetic and pharmacodynamic modelling [97].

Using the aforementioned senolytics, an open‑label interventional trial designed to reduce cellular senescence and improve frailty in adult survivors of childhood cancer is currently ongoing under the sponsorship of St. Jude Children's Research Hospital (Memphis, TN). The study evaluates short‑course senolytic regimens and is expected to reach its estimated completion date in 2027 (ClinicalTrials.gov Identifier: NCT04733534). The trial will compare two short‐duration senolytic regimens, that is, D+Q or fisetin alone, to improve walking speed and decrease senescent cell abundance in blood. Thus, in theory, combining senomorphics with senolytics, thanks to their complementary modes of action, that is, SASP modulation versus targeted senescent cell clearance (Table 1), could produce synergistic anti‐inflammatory effects while reducing off‐target cytotoxicity.

**TABLE 1:** Main drugs of the class of senolytic and senomorphics, their primary mechanism and key molecular targets, and cellular or clinical effects.

Senolytic vs. senomorphic drugs					
Class	Representative drugs	Primary mechanism	Key molecular targets	Effects on senescent cells	Systemic / Clinical relevance
**Senolytics**	Dasatinib + Quercetin (D+Q)	Tyrosine kinase inhibition + flavonoid‐mediated apoptosis	Src family kinases, ephrin receptors, PI3K	Induce apoptosis selectively in senescent cells	Reduced SASP, improved physical function, early clinical signals in IPF and frailty
	Fisetin	Flavonoid senolytic; mitochondrial stress sensitization	BCL‐2 family modulation, ROS signaling	Promotes apoptosis of senescent fibroblasts, endothelial cells	↓ SASP cytokines, improved vascular function (preclinical + early human data)
	Navitoclax (ABT‐263)	BCL‐2/BCL‐xL inhibition	BCL‐2 family	Strong senolytic activity in multiple tissues	Hematologic toxicity limits clinical use; potent in preclinical models
	FOXO4‐DRI peptide	Disrupts FOXO4–p53 interaction	FOXO4, p53	Triggers apoptosis in senescent cells	Reverses tissue dysfunction in mice; experimental
	HSP90 inhibitors	Proteostasis disruption	HSP90 chaperone network	Destabilizes survival pathways in senescent cells	Broad senolytic activity; toxicity concerns

Class of senomorphics	Representative drugs	Primary mechanism	Key molecular targets	Effects on senescent cells	Systemic/Clinical relevance
**JAK/STAT inhibitors**	Ruxolitinib, Baricitinib, Tofacitinib	JAK1/2 inhibition	JAK–STAT pathway	↓ IL‐6, IL‐8, MCP‐1; SASP suppression without killing cells	Used in older adults; preserve tissue integrity; strong senomorphic profile
**mTOR inhibitors**	Rapamycin, Everolimus	mTORC1 inhibition	mTORC1	↑ autophagy, improved proteostasis, ↓ SASP	Enhances vaccine responses, reduces inflammaging, improves metabolic resilience
**Metformin**	Metformin	AMPK activation; complex I modulation	AMPK, mitochondrial complex I	↓ ROS, ↓ IL‐6/TNF/CRP; SASP attenuation	Improves frailty indices, metabolic flexibility; major gerotherapeutic candidate
**NF‐κB inhibitors**	Aspirin, curcumin, bardoxolone	NF‐κB pathway suppression	IKK complex	↓ pro‐inflammatory SASP factors	Anti‐inflammatory effects across tissues; broad but nonspecific
**p38 MAPK inhibitors**	Losmapimod, pamapimod	p38 MAPK inhibition	MAPK14	↓ SASP transcription	Tested in muscle aging, chronic inflammation
**BET inhibitors**	JQ1, OTX015	Epigenetic modulation	BRD4	Blocks SASP gene expression	Potent SASP suppression; experimental
**CD38 inhibitors**	Compound 78c, daratumumab	NAD^+^ preservation	CD38	Restores NAD^+^ metabolism, reduces SASP	Relevant to metabolic aging and inflammaging

Parallel to senolytic approaches, senomorphic strategies seek to attenuate the pro‐inflammatory SASP without inducing cellular apoptosis. These interventions modulate transcriptional and metabolic pathways governing SASP expression, including the mechanistic target of rapamycin (mTOR), NF‐κB, JAK/STAT, and cGAS–STING signaling. Rapamycin and its derivatives (such as everolimus, sirolimus, and temsirolimus) are potent inhibitors of mTOR, and indeed they can boost immune responses [98, 99]. A recent systematic review has described the effects of mTOR inhibitors on age‐related physiological changes and diseases in adults [100]. A search across five databases yielded 18,400 articles, resulting in 19 studies included in the analysis. Overall, in most cases, mTOR inhibitors were able to decrease inflammation and improve parameters associated with aging in the immune, cardiovascular, and integumentary systems of healthy individuals or individuals with age‐related diseases, without significant effects on the endocrine, muscular, or neurological systems. Even if no serious adverse events were present in healthy donors, those with age‐related diseases displayed more infections and alterations of lipid metabolism, especially cholesterol. Notably, the lack of a clear relationship between the dose of mTOR inhibitors and the biological effect of the drug on different physiological systems indicates the need for more sophisticated studies.

Also, in a large group of patients (up to 77 years old) who were affected by myalgic encephalomyelitis/chronic fatigue syndrome, a low‐dose rapamycin was able to reduce postexertional malaise and other key symptoms, enhancing autophagic clearance and reducing chronic inflammation [101].

Metformin, via AMPK activation and mitochondrial complex I modulation, decreases ROS production and downregulates systemic inflammatory mediators, yielding improvements in metabolic resilience and frailty indices. Human clinical trials evaluating metformin as a gerotherapeutic include the landmark “Targeting Aging with Metformin” (TAME) program, the MET‑PREVENT randomized trial targeting frailty and physical performance, and the metformin in longevity (MILES) study assessing molecular biomarkers of aging. Even if these studies position metformin as a clinically advanced candidate for broad‑spectrum aging intervention, clinical data from the MET‐PREVENT trial show no improvements in measures of physical performance, activities of daily living, or quality of life in older people with sarcopenia and frailty [102]. Unfortunately, data on inflammatory or immune parameters were not available.

JAK inhibitors have demonstrated potent suppression of SASP‐associated cytokines, including IL‐6, IL‐8, and MCP‐1, across multiple cell types, while preserving tissue structural integrity through attenuation of inflammatory signaling and protection of extracellular matrix components [103]. Drugs like abrocitinib, baricitinib, and upadacitinib were mostly used in aged patients affected by chronic inflammatory diseases like atopic dermatitis, where they show clinical efficacy and a favorable safety profile [104]. Beyond dermatologic indications, JAK inhibitors have been extensively evaluated in older adults across inflammatory bowel diseases, rheumatoid arthritis, polymyalgia rheumatica, myelofibrosis, dermatomyositis, and acute inflammatory syndromes, consistently demonstrating potent suppression of the aforementioned cytokines, as well as improvements in functional outcomes and preservation of tissue integrity, underscoring their relevance as senomorphic agents in aging‐related inflammation.

Although short‐term studies indicate favourable tolerability profiles, long‐term safety remains an unresolved challenge. Potential risks—off‐target cytotoxicity, immune oversuppression, mitochondrial impairment, and unintended disruption of beneficial senescent cell populations (e.g., during wound healing)—underscore the necessity for highly selective drug delivery modalities and carefully calibrated dosing regimens. Ongoing work in nanoparticle targeting, prodrug strategies, and ligand‐directed senolytic delivery may help mitigate these concerns in future trials [105, 106, 107].

Taken together, current translational progress supports a shift toward multimodal, personalized therapeutics that integrate senolytics, senomorphics, metabolic modulators, and immune‐directed interventions within adaptive clinical trial architectures. Approaches that combine molecular biomarkers with functional and physiological endpoints are poised to accelerate the rigorous validation of safety and efficacy. Ultimately, these efforts aim not only to suppress inflammaging but to reconfigure the underlying biological networks that drive age‐associated decline, thereby enabling substantial and durable improvements in human healthspan.

## 6 Future Challenges in Targeting Inflammaging

**FIGURE 3:**
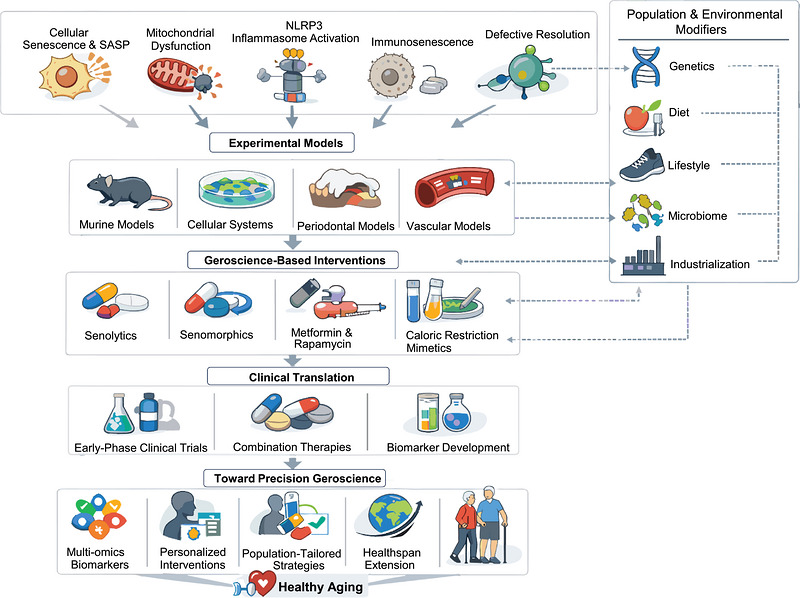
Recent advances and future directions in inflammaging. Emerging mechanisms of inflammaging have enabled the development of increasingly sophisticated experimental models and geroscience‐based interventions. These advances are fostering clinical translation and precision medicine approaches aimed at promoting healthy aging. Population and environmental modifiers, including genetics, diet, lifestyle, microbiome composition, and industrialization, influence all stages of the inflammaging trajectory.

Although recent years have brought substantial advances in defining the molecular landscape of inflammaging, as outlined in Figure 3, translating these insights into clinical practice remains a formidable challenge. A major hurdle lies in the identification of biomarkers that are not only robust and reproducible but also tissue‐specific and capable of capturing the dynamic and context‐dependent nature of age‐related inflammation. Conventional markers such as plasma levels of CRP, IL‐6, and TNF provide a general snapshot of systemic inflammatory status, yet fail to reflect the nuanced, tissue‐resolved, and dynamic senescence‐associated features that define the inflammaging phenotype.

The advent of high‐resolution technologies, including single‐cell transcriptomics, proteomics, metabolomics, and integrative multi‐omics, now enables unprecedented mapping of inflammatory trajectories across time and tissue compartments. These platforms hold promise for the development of dynamic biomarkers capable of tracking fluctuations in cytokine networks, senescence signatures, and metabolic states, thereby enhancing the prediction of functional decline and individual therapeutic responsiveness.

To bridge the gap between discovery and clinical utility, it will be essential to establish standardized biomarker panels that integrate soluble mediators, cellular phenotypes, and functional readouts, ensuring cross‐cohort reproducibility and translational relevance. In parallel, computational frameworks such as epigenetic, proteomic, and immune‐based “aging clocks” offer powerful tools to quantify biological age and aging velocity, potentially enabling stratification of aging trajectories and personalized intervention strategies. Recent sophisticated advances in immune‐based interventions targeting senescence have yielded promising results in both preclinical and translational settings. Anti‐urokinase plasminogen activator receptor (uPAR) CAR‐T cells have demonstrated the ability to selectively eliminate senescent cells in vitro and effectively clear precancerous and malignant cells in murine models of liver and lung pathology [108]. In aged mice, uPAR‐CAR‐T cell therapy also improved intestinal barrier integrity, enhanced mucosal regenerative capacity, modulated inflammatory responses, and reshaped gut immune and microbial homeostasis [109]. Despite the current challenges in translating these findings to humans, the studies offer clear proof of concept that targeted immune modulation can be leveraged to combat inflammaging.

Complementary findings have emerged from studies employing natural killer (NK) cell‐based approaches [110, 111, 112]. NK cell‐mediated clearance of senescent cells has been associated with lifespan extension in mice and appears to offer a favourable safety profile. In both aged murine models and human subjects, including healthy and obese individuals, adoptive NK cell infusion significantly reduced senescence‐associated markers and suppressed the SASP phenotype, without inducing overt toxicity [113]. These data collectively suggest that adoptive NK cell therapy may rejuvenate immunosenescence, promote the removal of senescent cells, and attenuate chronic low‐grade inflammation.

Stem cell‐based therapies have emerged as a promising strategy for combating aging‑related diseases. A substantial body of evidence indicates that both the number and functional capacity of multiple somatic stem and progenitor cell populations decline with age, leading to impaired tissue homeostasis and reduced regenerative potential across organs [114]. This progressive loss of stem cell fitness contributes to diminished repair responses, increased vulnerability to stress, and the gradual deterioration of physiological function that characterizes organismal aging [115, 116]. Clinical studies investigating stem cell‐based interventions in frail or aging patients have reported improvements in functional symptoms and reductions in circulating inflammatory markers following cell infusion [117]. However, these findings remain preliminary, often derived from small or heterogeneous cohorts, and require larger controlled trials to establish efficacy and long‑term safety.

Another major challenge concerns the safety and efficacy of therapeutic approaches. While senolytic and senomorphic therapies show promise in preclinical models, their long‐term safety, optimal dosing, and combinatorial strategies remain unresolved, especially in older adults with multimorbidity and polypharmacy. For example, caloric restriction mimetics, including glucagon‐like peptide‐1 (GLP‐1) analogs such as semaglutide, dulaglutide, liraglutide, exenatide, or lixisenatide, emulate or potentiate the physiological actions of endogenous GLP‐1. These agents enhance glucose‐dependent insulin secretion, delay gastric emptying, and modulate central appetite‐regulating pathways, collectively contributing to improved glycemic control and significant weight reduction [118]. Through their integrated metabolic and neuroendocrine effects, GLP‐1 agonists have emerged as promising tools in the management of metabolic dysfunctions and inflammation associated not only with obesity, but also with aging [119, 120]. In parallel, microbiome modulation is gaining increasing attention as a complementary anti‐inflammatory strategy [121]. Clearly, clinical applications of these combined approaches require careful evaluation due to the potential for unpredictable interactions with other therapeutic interventions [122].

Personalization represents another crucial frontier for modulating inflammaging, which is shaped by a unique interplay of genetic factors, microbiome composition, lifestyle, and environmental exposures [82]. The integration of multi‑omic data offers the possibility to identify individuals at highest risk, paving the way for targeted and truly personalized interventions. Combining dietary modifications, physical exercise, microbiome‐targeted actions, adequate sleep, stress‑management strategies, and pharmacological treatments may yield more balanced and effective outcomes [123]. In this context, machine learning and systems biology approaches hold considerable promise, as they enable the development of predictive models capable of guiding the design of individualized anti‑inflammaging therapies [124].

Finally, ethical, regulatory, and social considerations must accompany scientific and clinical progress. Interventions designed to promote healthy aging raise delicate questions about informed consent, long‐term safety monitoring, and fair access to treatments. On a broader level, issues such as potential lifespan extension, resource allocation, and intergenerational equity invite careful reflection on the societal implications of such advances. Tackling inflammaging effectively will require a truly multidisciplinary effort that integrates biomarker discovery, personalized medicine, combinatorial therapies, and population‐specific approaches, all supported by strong ethical and regulatory frameworks.

## Conflicts of Interest

The author declares no conflict of interest.

## Data Availability

Data sharing is not applicable to this article as no datasets were generated or analyzed during the current study.
